# β-amyloid accumulation enhances microtubule associated protein tau pathology in an APP^NL-G-F^/MAPT^P301S^ mouse model of Alzheimer’s disease

**DOI:** 10.3389/fnins.2024.1372297

**Published:** 2024-03-20

**Authors:** Lulu Jiang, Rebecca Roberts, Melissa Wong, Lushuang Zhang, Chelsea Joy Webber, Jenna Libera, Zihan Wang, Alper Kilci, Matthew Jenkins, Alejandro Rondón Ortiz, Luke Dorrian, Jingjing Sun, Guangxin Sun, Sherif Rashad, Caroline Kornbrek, Sarah Anne Daley, Peter C. Dedon, Brian Nguyen, Weiming Xia, Takashi Saito, Takaomi C. Saido, Benjamin Wolozin

**Affiliations:** ^1^Department of Anatomy and Neurobiology, Chobanian and Avedisian School of Medicine, Boston University, Boston, MA, United States; ^2^Department of Neuroscience, Center for Brain Immunology and Glia (BIG), School of Medicine, University of Virginia, Charlottesville, VA, United States; ^3^Department of Pharmacology, Physiology and Biophysics, Chobanian and Avedisian School of Medicine, Boston University, Boston, MA, United States; ^4^Department of Biological Engineering, Massachusetts Institute of Technology, Cambridge, MA, United States; ^5^Singapore-MIT Alliance for Research and Technology, Antimicrobial Resistance IRG, Campus for Research Excellence and Technological Enterprise, Singapore, Singapore; ^6^Department of Neurosurgical Engineering and Translational Neuroscience, Graduate School of Biomedical Engineering, Tohoku University, Sendai, Japan; ^7^LifeCanvas Technologies, Cambridge, MA, United States; ^8^Geriatric Research Education and Clinical Center, Bedford VA Healthcare System, Bedford, MA, United States; ^9^Department of Neurocognitive Science, Institute of Brain Science, Nagoya City University Graduate School of Medical Sciences, Nagoya, Japan; ^10^Laboratory for Proteolytic Neuroscience, RIKEN Center for Brain Science, Saitama, Japan; ^11^Department of Neurology, Chobanian and Avedisian School of Medicine, Boston University, Boston, MA, United States; ^12^Center for Systems Neuroscience, Boston University, Boston, MA, United States

**Keywords:** tauopathy, neurodegeneration, RNA binding proteins, tau oligomers, neuropathology, RNA methylation, neuritic plaques, clarity

## Abstract

**Introduction:**

The study of the pathophysiology study of Alzheimer’s disease (AD) has been hampered by lack animal models that recapitulate the major AD pathologies, including extracellular -amyloid (A) deposition, intracellular aggregation of microtubule associated protein tau (MAPT), inflammation and neurodegeneration.

**Methods:**

The humanized APP^NL-G-F^ knock-in mouse line was crossed to the PS19 MAPT^P301S^, over-expression mouse line to create the dual APPNL-G-F/PS19 MAPTP301S line. The resulting pathologies were characterized by immunochemical methods and PCR.

**Results:**

We now report on a double transgenic APP^NL-G-F^/PS19 MAPT^P301S^ mouse that at 6 months of age exhibits robust A plaque accumulation, intense MAPT pathology, strong inflammation and extensive neurodegeneration. The presence of A pathology potentiated the other major pathologies, including MAPT pathology, inflammation and neurodegeneration. MAPT pathology neither changed levels of amyloid precursor protein nor potentiated A accumulation. Interestingly, study of immunofluorescence in cleared brains indicates that microglial inflammation was generally stronger in the hippocampus, dentate gyrus and entorhinal cortex, which are regions with predominant MAPT pathology. The APP^NL-G-F^/MAPT^P301S^ mouse model also showed strong accumulation of N^6^-methyladenosine (m^6^A), which was recently shown to be elevated in the AD brain. m^6^A primarily accumulated in neuronal soma, but also co-localized with a subset of astrocytes and microglia. The accumulation of m^6^A corresponded with increases in METTL3 and decreases in ALKBH5, which are enzymes that add or remove m6A from mRNA, respectively.

**Discussion:**

Our understanding of the pathophysiology of Alzheimer’s disease (AD) has been hampered by lack animal models that recapitulate the major AD pathologies, including extracellular -amyloid (A) deposition, intracellular aggregation of microtubule associated protein tau (MAPT), inflammation and neurodegeneration. The APP^NL-G-F^/MAPT^P301S^ mouse recapitulates many features of AD pathology beginning at 6 months of aging, and thus represents a useful new mouse model for the field.

## Graphical abstract

This figure summarizes the pathophysiological changes occurring in the APP^NL-G-F^ /MAPT^P301S^ mouse model. This model has the humanized APP^NL-G-F^ gene as a knocking. The neurons consequently produce abundant Aβ_40/42_ which leads to rapid formation of extracellular Aβ plaques. The accumulation of Aβ stimulates inflammation and also feeds back to neurons to enhance MAPT pathology and neurodegeneration. The neurons over-express P301S MAPT, which leads to rapid formation of MAPT pathologies; the amount of pathology is enhanced modestly by the abundant production and aggregation of Aβ. The MAPT aggregation in the neurons stimulate the accumulation of cytoplasmic m^6^A, presumably as part of the translational stress response. Secreted MAPT stimulates microglial inflammation and astrocytosis. All of these factors accelerate neurodegeneration. The inflammation in this model tends to accumulate most in regions with MAPT pathology, particularly the hippocampus, although some reactive microglia are also observed near Aβ plaques.

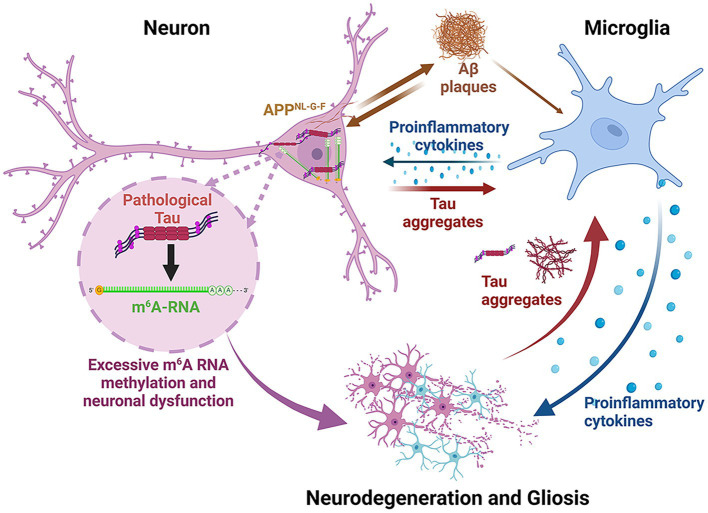

## 1 Introduction

The hallmark pathologies of Alzheimer’s disease (AD) consist of the accumulation of neuritic plaques composed of β-amyloid (Aβ), the accumulation of neurofibrillary tangles (NFTs) composed of microtubule associated protein tau (MAPT, Tau), inflammation and neurodegeneration (Scheltens et al., 2021). Mutations in amyloid precursor protein (APP) cause AD, however cognitive loss is only weakly correlated with the accumulation of Aβ (Scheltens et al., 2021). Cognitive loss is much more robustly correlated with MAPT based NFTs pathology, and mutations in MAPT are sufficient to cause dementia (frontotemporal dementia, FTD) (Scheltens et al., 2021). Mutations in MAPT might not cause AD because MAPT pathology does not drive the accumulation of Aβ pathology (Scheltens et al., 2021). The requirement of Aβ and MAPT pathologies to model AD has posed a challenge for mouse models of AD because mutations in either gene alone are insufficient to produce both pathologies in mice.

Models relying only on genetic modification of amyloid precursor protein (APP) develop abundant Aβ plaques but produce little MAPT pathology beyond modest increases in phosphorylation (Sasaguri et al., 2022). Mouse models over-expressing mutant APP, such as Tg2576, or over-expressing mutant APP and mutant presenilin 1, such as 5xFAD, rapidly develop accumulated Aβ and develop neuritic plaques. However, over-expressing APP and presenilin 1 cause effect resulting from the over-expression that are unrelated to the disease process (Saito et al., 2014; Sakakibara et al., 2018). These problems have been addressed by knocking in the human APP gene containing mutations that increase production of Aβ40 and/or Aβ42 (Saito et al., 2014; Sakakibara et al., 2018). These mice develop robust plaque pathology beginning as early as 3 months of age, however they develop little tau pathology and little neurodegeneration (Saito et al., 2014; Sakakibara et al., 2018).

The absence of robust MAPT pathology in mouse models expressing only endogenous MAPT likely derives from the low aggregation propensity of murine MAPT (Eckermann et al., 2007; Mocanu et al., 2008). This limitation has been addressed by introducing human tau constructs into the mouse brain (Hutton et al., 2001; Sasaguri et al., 2022). Many mouse models have been developed based on over-expressing wild type (WT) tau or mutant forms of tau linked to frontotemporal dementia (Hutton et al., 2001; Sasaguri et al., 2017, 2022). These models develop robust tau pathologies, including pathologically phosphorylated, misfolded, oligomeric and/or fibrillar forms of tau pathology. These models also exhibit progressive neurodegeneration, which is consistent with observations in humans that cognitive loss is more closely associated with tau pathology than Aβ pathology (Lewis et al., 2000; Ramsden et al., 2005; Polydoro et al., 2009). Human MAPT knock-in models have also been developed, and these models produce tau pathology, but only very late in the murine life span (~15 months) (Saito et al., 2014; Sakakibara et al., 2018; Sasaguri et al., 2022).

Multiple groups have explored crossing transgenic APP mouse over-expression models with tau mouse models (Ribe et al., 2005; Saul et al., 2013; Chabrier et al., 2014; Heraud et al., 2014; Stancu et al., 2014; Pooler et al., 2015; Chen et al., 2016; Kang et al., 2021). The results generally show that Aβ accelerates tau pathology (Chabrier et al., 2014; Heraud et al., 2014; Stancu et al., 2014; Chen et al., 2016). The tau pathology does not appear to increase Aβ deposition (Saul et al., 2013; Chabrier et al., 2014; Heraud et al., 2014; Stancu et al., 2014; Chen et al., 2016; Kang et al., 2021); indeed, in some cases it accelerates removal of Aβ (Chen et al., 2016). These studies all suffer because they exhibit artifacts arising from the over-expression APP (WT or mutant) and in some cases mutant presenilins (Balducci and Forloni, 2011; Saito et al., 2014; Sasaguri et al., 2017). Recent studies have begun to explore crossing human knockin models (Sasaguri et al., 2022). Human APP knockin (KI) models develop robust Aβ pathology, yet do not exhibit artifacts associated with APP over-expression, such as elevated levels of APP cleavage products (Saito et al., 2014). Human MAPT KI models exhibit delayed tau pathology, as does a cross between the two KI models (Saito et al., 2019; Islam et al., 2023). Thus, we sought to create a model that avoided artifacts associated with APP over-expression, yet utilizes over-expression of P301S MAPT to promote robust tau pathology.

We now report creating a mouse model in which the APP^NL-G-F^ KI and the PS19 P301S MAPT mouse lines were crossed (Yoshiyama et al., 2007; Saito et al., 2014). The resulting mouse model, termed APP^NL-G-F^/MAPT^P301S^, develops many aspects of AD pathology. The APP^NL-G-F^/MAPT^P301S^ mouse exhibits a progressive increase in Aβ load, neuritic plaques, all major forms of tau pathology, as well as exhibiting enhanced microglial activation, astrogliosis, neurodegeneration and a progressive loss in cognitive function. This model also exhibits other key elements of AD pathology including inflammation and elevated levels of N^6^-methyl-adenosine (m^6^A) tagged RNA, which has recently been shown to change strongly with disease progression (Jiang L. et al., 2021). In this APP^NL-G-F^/MAPT^P301S^ model, we also found that Aβ pathology potentiates MAPT pathology, astrogliosis, inflammation and neurodegeneration. MAPT pathology does not appear enhance the accumulation of Aβ pathology or inflammation beyond that observed in the APP^NL-G-F^ mouse, although the inflammation that did occur tended to be in regions with predominant MAPT pathology. Intriguingly we found that levels of m^6^A RNA correlate with MAPT but not Aβ pathology.

## 2 Methods

### 2.1 Mice

Use of all animals was approved by the Boston University Institutional and Animal Care and Use Committee (IACUC). All animals used in this study were handled according to IACUC approved protocols and housed in IACUC approved vivariums at the Boston University Animal Science Center. The APP^NL-G-F^ mouse model was generated by Saito et al. (2014) at the RIKEN Brain Science Institute in Japan; the PS19 (B6; C3-Tg (Prnp-MAPT*P301S)PS19Vle/J, stock #008169) and C57BL/6J (stock #000664) mice were originally purchased from the Jackson Laboratory in Maine (Yoshiyama et al., 2007). All mice used were on a congenic C57BL/6 J background. To generate the APP^NL-G-F^/MAPT^P301S^ cross, homozygous APP^NL-G-F^ mice were bred with heterozygous PS19 mice resulting in either the double transgenic cross or heterozygous APP^NL-G-F^ mice. Due to littermates being heterozygous for the APP mutations, wild-type C57BL/6 J mice were used as controls.

### 2.2 Immunoblot

The homogenized lysate for western blot were collected from fresh frozen brain tissue with RIPA lysis buffer. Reducing and non-reducing protein samples were separated by gel electrophoresis and transferred to 0.2 μm nitrocellulose membranes using the Bolt SDS-PAGE system (Life Technologies). Membranes were blocked in 5% nonfat dry milk (NFDM) in PBS supplemented with 0.025% Tween-20 (PBST) for 1 h RT, followed by incubation overnight at 4°C in primary antibody diluted in 5% bovine serum albumin/PBST. Primary antibodies used were as follows: pTau217 (1, 500) anti-tau antibody (rabbit, Thermo Scientific, Cat# 44744); 4G8, Anti-Amyloid β Antibody, clone W0-2, reactive to amyloid-β, aa 17–24 (Millipore Sigma, Cat# MABN10). Membranes were then washed 3 times with PBST and incubated in HRP-conjugated secondary antibodies (Jackson ImmunoResearch) diluted in 1% BSA/PBST at RT for 1 h. After incubation in secondary antibody, membranes were washed 3 times in PBST and developed using SuperSignal West Pico Chemilluminescent ECL substrate (Thermo Fisher Scientific, cat# 34080).

### 2.3 Immunohistochemistry

Wild type (WT), APP^NL-G-F^, MAPT^P301S^, and APP^NL-G-F^/MAPT^P301S^ mouse brains were collected at 3, 6, and 9 months of age, respectively. Briefly, mice were anaesthetized with isoflurane and then the hearts perfused with 20 mL ice cold PBS for 5 min followed by perfusion with 20 mL ice cold 4% PFA for 10 min. The mouse brains were dissected and placed in 4% PFA on ice for 2 h. Then the brains were washed with PBS and transferred into 30% sucrose/PBS until the brains sank to the bottom of the tube (about 48 h), and sectioned. The fixed brains were sliced into 30 μm coronal sections by cryostat, and stored in 0.005% sodium azide/PBS solution at 4°C for up to 3 months. For long-term storage, the sections were transferred into cryoprotectant solution (30% glycerol and 30% ethylene glycol in PBS), and stored at −20°C.

For immuno-labeling, the 30 μm free-floating sections with hippocampus or lateral entorhinal cortex (LEnt) were blocked with 5% BSA and 5% goat serum in PBST (PBS/0.25% Triton X-100) for 30 min and then incubated with monoclonal 6E10 antibody (BioLegend, cat# 803001, 1:1000 dilution) overnight at 4°C. On the second day, sections were washed with PBST three times and then incubated with biotinylated goat anti-mouse IgG antibody (Vector Laboratories, cat# BA-9200) for 2 h RT. The antibody binding was visualized using a Vectastain ABC Kit (Vector Laboratories, cat# PK-6100) and diaminobenzidine (DAB) substrate tablet (Sigma-Aldrich, cat# D4293-50SET) as described previously (Jiang et al., 2019). Images were captured by Keyence microscope bz-x800.

### 2.4 Immuno-fluorescence staining of fixed brain tissues

For immuno-fluorescence labeling, selected sections of hippocampus from bregma-1.8 and LEnt from bregma-2.8, were washed in PBS for 10 min and then permeabilized in 0.5 mL PBS/0.25% Triton X-100 (PBST). The tissues were blocked in PBST supplemented with 5% BSA and 5% normal donkey serum for 1.5–2 h at RT. The 1° antibodies were diluted in 5% BSA/PBST, added to the sections and incubated overnight at 4°C. On the second day, the sections were washed 3× in PBST, 15 min each. Next the sections were incubated for 2 h at RT in 5% BSA/PBST containing 2° antibodies (1:700 for Dylight-/Alexa-conjugated antibodies made in donkey, Thermo Fisher Scientific). For DAPI nuclei stain, the sections were incubated for 15 min in DAPI (1:10000)/PBST, then washed 2× with PBST and 1× with PBS, 10 min each. The labeled brain sections were mounted onto microscope glass slides in Prolong gold anti-fade reagent. The following 1° antibodies were used in this study: NeuN (chicken, Millipore, cat# ABN91), 1:300; MC1 (mouse, provided by Peter Davies, Northwell), 1:100; TOMA2 (mouse, provided by Rakez Kayed, UTMB Galveston), 1:200 (Castillo-Carranza et al., 2014); 6E10 antibody (BioLegend, cat# 803001, 1:1000 dilution); 4G8, Anti-Amyloid β Antibody, clone W0-2, reactive to amyloid-β, aa 17–24 (Millipore Sigma, Cat# MABN10), 1:1000; Iba-1 (Abcam, Cat# ab5076), 1:1000; Images were captured by Carl Zeiss confocal LSM700.

### 2.5 Tissue preservation and clearing, Immunolabeling and imaging

Paraformaldehyde-fixed samples were preserved with using SHIELD reagents (LifeCanvas Technologies) using the manufacturer’s instructions (Park et al., 2018). Samples were delipidated using LifeCanvas Technologies Clear+ delipidation reagents. Following delipidation samples were labeled using eFLASH (Costa et al., 2019) technology which integrates stochastic electrotransport (Kim et al., 2015) and SWITCH (Murray et al., 2015), using a SmartBatch+ (or SmartLabel) device (LifeCanvas Technologies). After immunolabeling, samples were incubated in 50% EasyIndex (RI = 1.52, LifeCanvas Technologies) overnight at 37°C followed by 1 day incubation in 100% EasyIndex for refractive index matching. After index matching the samples were imaged using a SmartSPIM axially-swept light sheet microscope using a 3.6x objective (0.2 NA) (LifeCanvas Technologies).

### 2.6 Measurements of pathological proteins Aβ and phosphorylated tau by enzyme-linked immunosorbent assay

The Measurements of pathological Aβ and phosphorylated tau by enzyme-linked immunosorbent assay (ELISA) was performed as described previously (Xia et al., 2009; Stein et al., 2015; Stathas et al., 2022). In brief, frozen mouse brain tissue was homogenized in 5:1 volume of freshly prepared, ice cold 5 M guanidine hydrochloride in Tris-buffered saline (20 mM Tris-HCl, 150 mM NaCl, pH 7.4), which contained 1:100 Halt protease inhibitor cocktail (Thermo Fisher Scientific) and 1:100 phosphatase inhibitor cocktail 2 & 3 (Sigma-Aldrich) as previously reported (Park et al., 2018; Costa et al., 2019). The homogenate was then shaken (regular rocker) overnight at room temperature. The lysate was diluted with 1% Blocker A [Meso Scale Discovery (MSD), #R93BA-4] in wash buffer according to specific immunoassays: 1:4000 for Aβ_1–38_, Aβ_1–40_, and Aβ_1–42_ (MSD #K15200E-2), and 1:300 for p-tau_181_, p-tau_202_ (MSD custom kit), total tau and p-tau_231_ (MSD #K15121D-2). Samples were centrifuged at 17,000 g and 4°C for 15 min. The supernatant was subsequently applied to the immunoassays, and the original homogenate was aliquoted and stored at −80°C.

To capture MAPT phosphorylated at Thr residue 181, antibody AT270 was used. The detecting antibody was the biotinylated HT7 that recognizes residue 159–163 of tau (Thermo Fisher Scientific). To measure p-tau^396^, a rabbit monoclonal antibody against p-tau^396^ (Abcam, ab156623) was used as the capturing antibody, and HT7 was used as a detecting antibody. Sulfo-tag conjugated streptavidin secondary antibody was used for signal detection by the MSD platform. MSD SECTOR Imager 2400 was used to measure p-tau^396^ levels. Internal calibrators of p-tau and tau were used (MSD). p-tau levels were measured in arbitrary units, which may or may not be related among the different epitopes. Standards with known concentrations were used for Aβ, and all standards and samples were run in duplicate. Measurements were made using the multi-detection SPECTOR 2400 Imager (MSD).

### 2.7 Reverse transcription quantitative real-time PCR

The reverse transcription quantitative real-time PCR (RT-qPCR) was applied for the rapid detection of gene expression changes of pro-inflammatory cytokines and complement proteins in the brain tissue of WT, APP^NL-G-F^, MAPT^P301S^, and APP^NL-G-F^/MAPT^P301S^ double transgenic, respectively. The primers used in this study are listed below:

**Table 1:** 

Internal control	Mouse GAPDH	Forward primer	CAACAGCAACTCCCACTCTTC
		Reverse primer	GGTCCAGGGTTTCTTACTCCTT
Pro-inflammation	Mouse TNF-α	Forward primer	GACCCTCACACTCAGATCATCTTCT
		Reverse primer	CCTCCACTTGGTGGTTTGCT
Pro-inflammation	Mouse IL-1β	Forward primer	GAAGAGCCCATCCTCTGTGA
		Reverse primer	TTCATCTCGGAGCCTGTAGTG
DAM microglia	Mouse TREM2	Forward primer	GACCTCTCCACCAGTTTCTCC
		Reverse primer	TACATGACACCCTCAAGGACTG
Neurotrophic factor	Mouse BDNF	Forward primer	AGGCAACTTGGCCTACCCAGGTGTG
		Reverse primer	TACTGTCACACACGCTCAGCTCCCC

### 2.8 Images analysis

The intensity in immuno-fluorescence or DAB stained brain sections were measured by MATLAB program; the NeuN-positive cell number were quantified by Image J automatically cell counting. The quantification of cell numbers was done in a blinded manner, with the investigator analyzing the samples being different than the investigator coding the samples.

Studies using the Imaris Bitplane program were used to create videos of immunofluorescence in the cleared brains.

### 2.9 Statistical analysis

Statistical analyses and figures artwork were performed using GraphPad Prism version 9.00 for Windows with two sided α of 0.05. All group data are expressed as mean ± SEM. Colum means were compared using one-way ANOVA with treatment as the independent variable. And group means were compared using two-way ANOVA with factors on genotype and age time course of the mice, respectively. When ANOVA showed a significant difference, pair wise comparisons between group means were examined by Tukey’s multiple comparison test. Significance was defined at *p* < 0.05.

## 3 Results

### 3.1 Generation of the APP^NL-G-F^/MAPT^P301S^ mouse line

**Figure 1:**
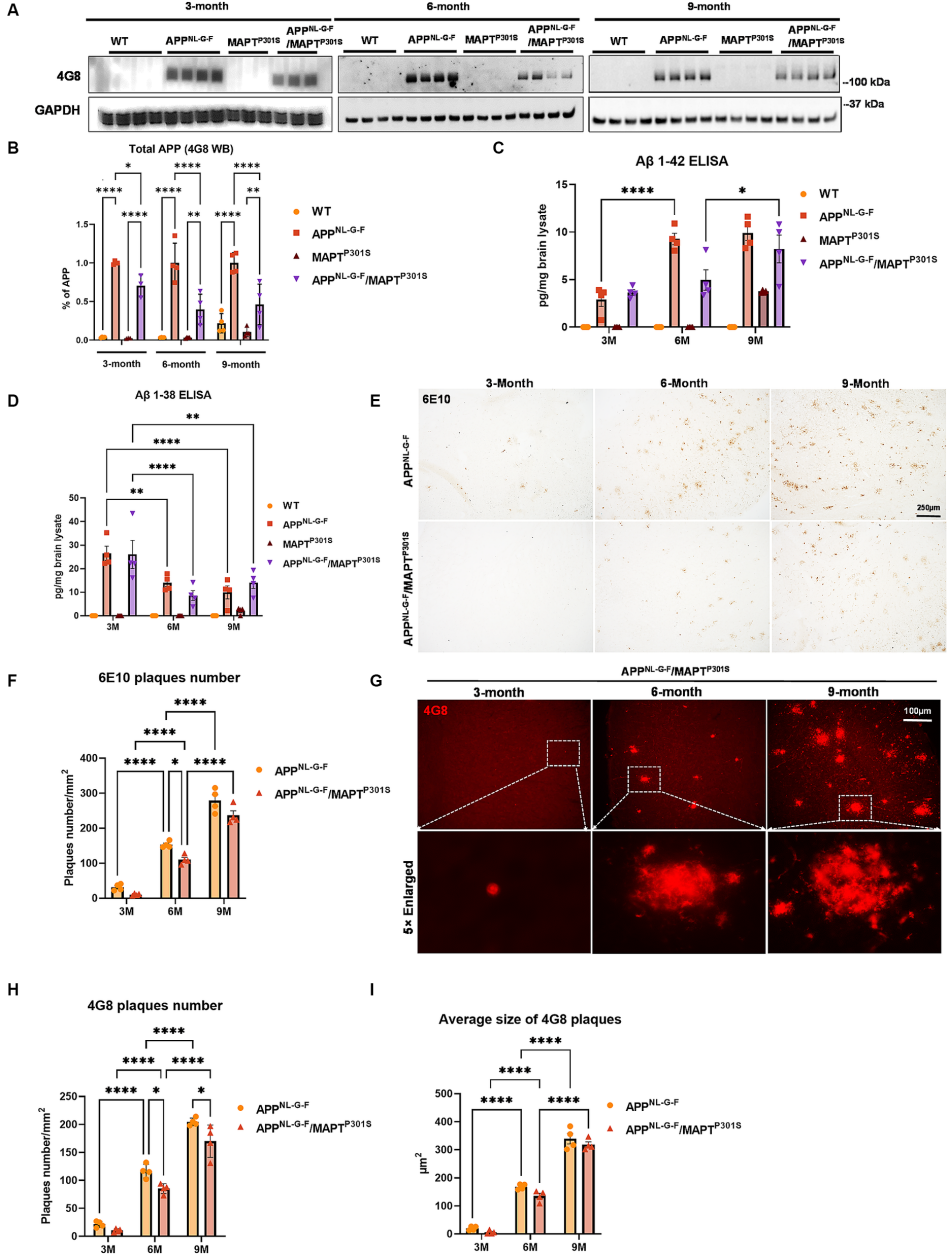
Beta-amyloid deposition accumulates in a time-dependent manner in the APP^NL-G-F^/MAPT^P301S^ mouse. **(A)** Representative images of immunoblot with 4G8 antibody showed the expression of human amyloid precursor protein (APP) in the APP^NL-G-F^ and APP^NL-G-F^/MAPT^P301S^ mouse brain but not wild type (WT) or MAPT^P301S^ brain. Total brain lysates were harvested at 3, 6, and 9 months for each of the four mouse genotypes (WT, APP^NL-G-F^, MAPT^P301S^, and APP^NL-G-F^/MAPT^P301S^ double transgenic, respectively). GAPDH was detected as the internal control. **(B)** Quantification of human APP expression in the total brain lysate as shown in **(A)**. *N* = 4, data shown as mean ± SEM. **(C,D)** The amount of Aβ_38_ and Aβ_42_ in the total brain lysate detected by V-PLEX Aβ Peptide Panel 1 (4G8) Kit. Brain lysate from 4 genotypes of mice were detected at 3, 6, and 9 months, respectively. *N* = 4 mice in each condition, data shown as mean ± SEM. Two-way ANOVA with Tukey’s multiple comparisons test, ^*^p < 0.05, ^**^p < 0.01, and ^****^p < 0.001. **(E)** The 6E10 antibody (reactive to aa 1–16 Aβ and to APP) was used to examine the diffused amyloid plaques in the aging process of APP^NL-G-F^ and APP^NL-G-F^/MAPT^P301S^ mouse brain. Representative DAB staining images showed the progressive increase of 6E10 positive β-amyloid plaques in the entorhinal cortex from 3 to 6 and 9 months of mouse brain. Scale bar 250 μm. **(F)** Quantification for the number of 6E10 positive β-amyloid plaques averaged over 1 mm^2^ squares across each brain slice. *N* = 4 mice in each group, data shown as mean ± SEM. Two-way ANOVA with Tukey’s multiple comparisons test, ^*^p < 0.05 and ^****^p < 0.001. **(G)** The 4G8 antibody (reactive to Aβ, aa 17–24) was used to examine the compact amyloid plaques in the aging process of APP^NL-G-F^ and APP^NL-G-F^/MAPT^P301S^ mouse brain. Representative red fluorescence labeling stacked images showed the progressive increase of 4G8 positive β-amyloid plaques in the entorhinal cortex from 3 to 6 and 9 months of mouse brain. Scale bar 100 μm. **(H,I)** Quantification of the number and average size of 4G8 positive Aβ + plaques. *N* = 4 mice per group, 3 sections were used for each mouse. Data shown as mean ± SEM. Two-way ANOVA with Tukey’s multiple comparisons test, ^*^p < 0.05 and ^****^p < 0.001.

The APP^NL-G-F^ mouse line was developed to avoid artifacts associated with over-expressing APP (Saito et al., 2014). These mice develop robust neuritic plaque pathology. We initiated the project by crossing homozygous APP^NL-G-F^ mice with heterozygous P301S MAPT mice. The mice generated normal mendelian patterns of inheritance, producing expected genotypes in the offspring. The resulting mouse lines were aged, harvested and examined patterns of APP expression, as well as the accumulation of Aβ and neuritic plaques at 3, 6, and 9 months of age. Immunoblots of APP using the 48G antibody showed that expression of the tau transgene reduces the expression of APP at each age (Figures 1A,B).

To explore the accumulation of extracellular Aβ in the transgenic mice, we examined the amount of Aβ_38_ and Aβ_42_ in the total brain lysates by V-PLEX Aβ Peptide ELISA Kit. The V-PLEX platform offers analytically validated singleplex and multiplex assay kits, which can provide accurate and reproducible results with consistency from lot to lot (van Dyck et al., 2023). ELISA quantification of Aβ_42_ showed that Aβ_42_ levels progressively increased with age in both APP^NL-G-F^ and APP^NL-G-F^ x MAPT^P301S^ mouse brains (Figure 1C). Notably, in the APP^NL-G-F^ mouse the Aβ_42_ level peaked at 6 months and remained constant at 9 months while levels of Aβ_42_ steadily increased with age (at 3, 6, and 9 months) in APP^NL-G-F^ × MAPT^P301S^ mouse brain (Figure 1C). In contrast, Aβ_38_ levels decreased with age in APP^NL-G-F^ and APP^NL-G-F^ × MAPT^P301S^ mice (Figure 1D).

Quantification of Aβ plaque-load by immunohistochemistry indicated that plaque load results largely followed the quantification of Aβ_42_ determined by ELISA. Mice at 3, 6, and 9 months were harvested and subjected to immunohistochemistry using both colorimetric and fluorescent approaches. Analysis of sections with antibody 6E10, which preferentially detects diffuse plaques, was done using the colorimetric DAB/peroxidase method. These results showed a progressive increase in amyloid plaque accumulation in the APP^NL-G-F^ mouse line as well as the APP^NL-G-F^/MAPT^P301S^ mouse line (Figures 1E,F). Importantly, amyloid plaques showed age-dependent increases for both the APP^NL-G-F^ and the APP^NL-G-F^/MAPT^P301S^ mouse lines (Figures 1E–I), although the APP^NL-G-F^/MAPT^P301S^ mouse line exhibited less accumulation than for the APP^NL-G-F^ mouse line (Figures 1E–G). For instance, a robust, progressive increase in 4G8-positive plaque load was observed in both the APP^NL-G-F^ mouse line as well as the APP^NL-G-F^ × MAPT^P301S^ mouse line, but the APP^NL-G-F^ × MAPT^P301S^ mice exhibited fewer (15–20%) plaques than the APP^NL-G-F^ mice at 6 and 9 months (Figures 1G,H). Interestingly, the average plaque size did not differ between the two groups (Figures 1G,I).

These results indicate that the APP^NL-G-F^ × MAPT^P301S^ double transgenic mouse model recapitulates the progressive accumulation Aβ plaques and levels similar to that seen in the APP^NL-G-F^ model although total levels of APP and Aβ accumulation are lower in the cross than with the knock-in gene alone.

### 3.2 APP^NL-G-F^ potentiates progression of MAPT pathology in the APP^NL-G-F^/MAPT^P301S^ double transgenic mice

Another goal of the APP^NL-G-F^/MAPT^P301S^ double transgenic mice is to recapitulate the development of MAPT pathology associated with cognitive decline in AD patients. To investigate the MAPT aggregation in the MAPT^P301S^ and APP^NL-G-F^/MAPT^P301S^ mice as well as the effect of APP^NL-G-F^ on MAPT pathology, we examined MAPT phosphorylation, oligomerization and misfolding in the aging process of all four genotypes, including MAPT^P301S^, APP^NL-G-F^ and APP^NL-G-F^/MAPT^P301S^ in comparison to WT C57BL/6 control. The total brain lysates were prepared from the fresh frozen brain tissue harvested at 3, 6, and 9 months and homogenized in RIPA buffer.

**Figure 2:**
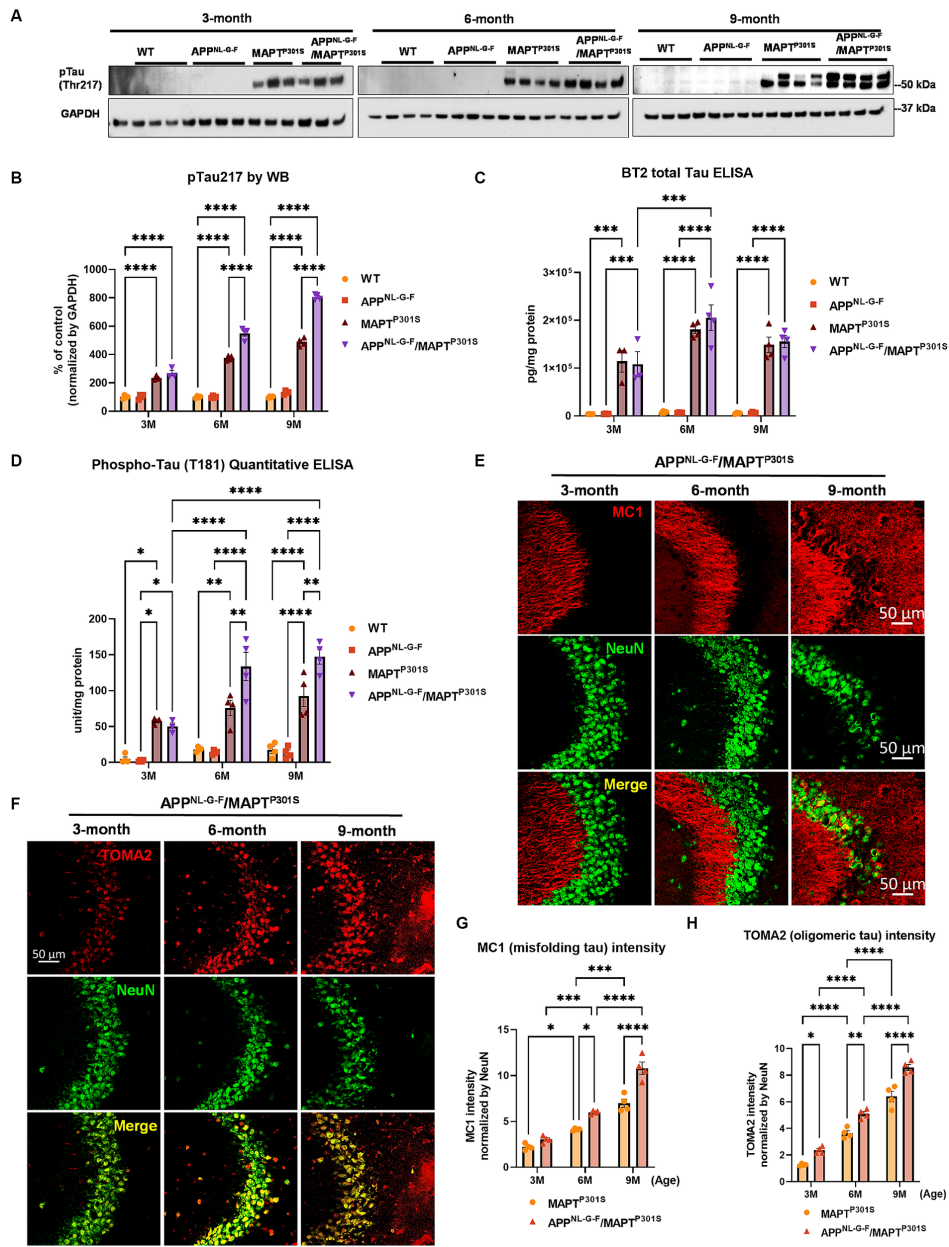
APP^NL-G-F^ potentiates progression of MAPT pathology in the APP^NLGF^/MAPT^P301S^ double transgenic mice. **(A)** Representative images of western blot with phosphorylated tau antibody on phosphor-site threonine217 (pTau217) showed the accumulation of hyper phosphorylated tau in the MAPT^P301S^ and APP^NL-G-F^/MAPT^P301S^ mouse brain but not wild type (WT) or APP^NL-G-F^ brain. Total brain lysate were harvested at 3, 6, and 9 months for each of the four genotypes (WT, APP^NL-G-F^, MAPT^P301S^, and APP^NL-G-F^/MAPT^P301S^) of mice, respectively. GAPDH was detected as the internal control. **(B)** Quantification of pTau217 in the total brain lysate as shown in **(A)**. *N* = 4 mice in each group, data shown as mean ± SEM. Statistics was by two-way ANOVA with post hoc Tukey’s multiple comparisons test, ^****^p < 0.001. **(C,D)** Detection of total MAPT levels in the brain lysates with the BT2 antibody (epitope between aa 194–198, but not PHF tau) and threonine181 phosphorylated tau (pTau181) by AT270 antibody, respectively, with ELISA assay. *N* = 4 mice in each group, data shown as mean ± SEM. Statistical analysis was by two-way ANOVA with post hoc Tukey’s multiple comparisons test, ^***^p < 0.005 and ^****^p < 0.001. **(E)** Representative fluorescence labeling images showed the accumulation of misfolding tau (by MC1 antibody, red) in the hippocampal CA3 region of APP^NL-G-F^/MAPT^P301S^ mice over 3, 6, and 9 months. NeuN antibody (green) was used to label the neuronal cells. Scale bar 50 μm. **(F)** Representative fluorescence images show the accumulation of oligomeric tau (TOMA2 antibody, red) in the hippocampal CA3 brain region of APP^NL-G-F^/MAPT^P301S^ mice over 3, 6, and 9 months. NeuN antibody (green) was used to label neurons. Scale bar 50 μm. **(G,H)** Quantification of misfolded tau (MC1) and oligomeric tau (TOMA2) as shown in **(E,F)** respectively. Total fluorescence intensity was collected and then normalized by NeuN for statistics. *N* = 4 mice in each group, data is shown as mean ± SEM. Two-way ANOVA was used for statistics followed by post hoc analysis with Tukey’s multiple comparisons test, ^*^p < 0.05, ^**^p < 0.01, ^***^p < 0.005, and ^****^p < 0.001.

Recent studies highlight phosphorylated MAPT at threonine MAPT 217 (pTau217) as a new promising plasma biomarker for pathological changes implicated in AD (Telser et al., 2022). Immunohistochemistry with postmortem AD brain tissue also demonstrated that pTau217 is found in neurofibrillary tangles (NFTs) and neuropil threads that are also positive for pTau181, 202, 202/205, 231, and 369/404 (Wennstrom et al., 2022). Levels of pTau217 also correlate with total Aβ and NFT brain load in AD brain (Ashton et al., 2022; Wennstrom et al., 2022). Thus, we use levels of pTau217 as biomarker for evaluating the pathological progression of the APP^NL-G-F^/MAPT^P301S^ mouse model. Immunoblots of brain lysates from MAPT^P301S^ and APP^NL-G-F^/MAPT^P301S^ mice showed progressive accumulation of pTau217 over 3, 6, and 9 months range (Figures 2A,B). The APP^NL-G-F^/MAPT^P301S^ exhibited significantly more pTau217 MAPT phosphorylation at 6 and 9 months compared to MAPT^P301S^ alone, which suggest that APP^NL-G-F^ potentiated MAPT phosphorylation (Figures 2A,B). Total levels of MAPT were measured using the BT2 antibody (epitope between aa 194–198, but not PHF MAPT) by ELISA assay. We also used ELISA assays to measure levels of hyper-phosphorylated MAPT in the brain lysate, quantifying MAPT phosphorylation at threonine181 (pTau181) with the AT270 antibody (Figures 2C,D). The result showed that both APP^NL-G-F^/MAPT^P301S^ and MAPT^P301S^ accumulated hyperphosphorylated tau, but crossing APP^NL-G-F^ to MAPT^P301S^ increased the accumulation of pTau181 and 217 (Figures 2B–D).

In addition to the MAPT phosphorylation, we also examined the MAPT misfolding by the conformational tau marker MC1 (epitope within aa 312–322) with immuno-fluorescence labeling. Our result showed that MAPT tau misfolding started at the dendritic compartment of the neurons in CA3 when the mice were 3 months old (Figure 2E). By 9 months of age, the misfolded MAPT had also distributed and accumulated in neuronal soma (Figure 2E). Compared to MAPT^P301S^ alone, the APP^NL-G-F^/MAPT^P301S^ double transgenic mouse consistently potentiated accumulation of misfolded MAPT over the time span of 3, 6, and 9 months of age (Figure 2G).

Studies suggest that MAPT oligomers are the more toxic species that induce neurodegeneration (Lasagna-Reeves et al., 2011; Castillo-Carranza et al., 2014; Apicco et al., 2018; Jiang et al., 2019, 2020). To assess the assembly of MAPT oligomers in the APP^NL-G-F^/MAPT^P301S^ double transgenic mouse, we detected MAPT by immuno-fluorescence in hippocampus using the antibody TOMA2, which specifically recognizes oligomeric MAPT (Sengupta et al., 2018). The result showed strong labeling of oligomeric MAPT that was selective for the somatic compartment of the neurons and exhibited progressive age dependent accumulation as the mice (Figure 2F). Quantification of TOMA2 intensity revealed that MAPT oligomers were more abundant in APP^NL-G-F^/MAPT^P301S^ double transgenic mouse compared to MAPT^P301S^ (Figure 2H).

These results demonstrate that APP^NL-G-F^ potentiates the progression of MAPT pathology including phosphorylation, mis-conformation and oligomerization in the APP^NL-G-F^/MAPT^P301S^ double transgenic mice.

### 3.3 APP^NL-G-F^ is the predominant driver of microglial activation and astrogliosis

**Figure 3:**
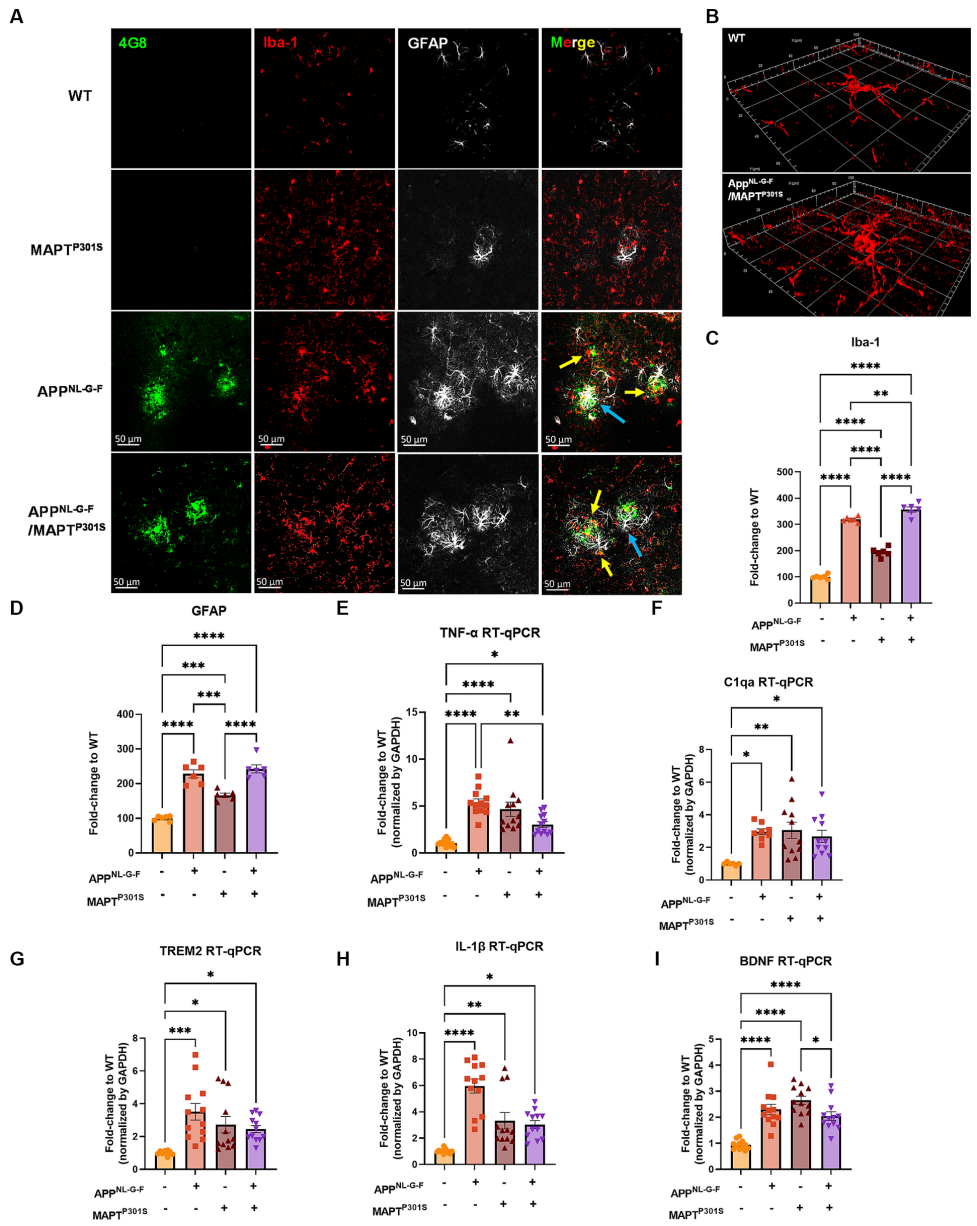
APP^NL-G-F^ is the predominant driver of microglial activation and astrogliosis. **(A)** Representative fluorescence labeling images showed the activation and morphological changes of microglia (by Iba-1 antibody, red) in the frontal cortex in pathological APP^NL-G-F^ and/or MAPT^P301S^ mouse brain at 9 months old. Astrocytes (GFAP antibody, white) were robustly activated around Aβ plaques. Scale bar 50 μm. **(B)** Enlarged image showed the amoeba-like morphological changes of microglia in the APP^NL-G-F^/MAPT^P301S^ mouse brain. **(C,D)** Quantification of microglial activation by Iba-1 intensity and astrogliosis by GFAP intensity as shown in **(A)**. Data was normalized to the fold increase of WT control. *N* = 6 mice in each group, data is shown as mean ± SEM. Two-way ANOVA was used for statistics followed by post hoc analysis with Tukey’s multiple comparisons test, ^**^p < 0.01, ^***^p < 0.005, and ^****^p < 0.001. **(E–I)** Quantification on the transcriptomic levels of inflammatory factors in the brain of WT, APP^NL-G-F^, MAPT^P301S^, and APP^NL-G-F^/MAPT^P301S^ mouse lines, respectively, at 9 months. The pro-inflammatory factors TNF-α, IL-1β, C1qa as well as BDNF were quantified by RT qPCR. Results are shown as fold-change vs. WT control. *N* = 10–12 mice per group; data is shown as mean ± SEM. One-way ANOVA was used for statistics followed by post hoc analysis with Tukey’s multiple comparisons test, ^*^p < 0.05, ^**^p < 0.01, ^***^p < 0.005, and ^****^p < 0.001.

Microglial activation and astrogliosis are thought to be induced by Aβ and MAPT pathology, and contribute to subsequent neurodegeneration in AD (Hansen et al., 2018; Leng and Edison, 2021). To characterize glial activation in the APP^NL-G-F^/MAPT^P301S^ double transgenic mouse, we analyzed the microglial and astrocytic morphologies as well as levels of inflammatory factor transcripts during the aging process in the APP^NL-G-F^/MAPT^P301S^ mouse brain. Our data showed that microglia exhibit a ramified appearance under basal conditions labeled by Iba-1 marker. The presence of the APP^NL-G-F^ gene induced morphological changes with thickening processes and amoeboid shape (Figures 3A,B). The morphological changes induced by the APP^NL-G-F^ gene were similar for the APP^NL-G-F^ mouse and the APP^NL-G-F^/MAPT^P301S^ mouse lines (Figure 3A). Quantification of these morphological changes suggested that the effect of the APP^NL-G-F^ gene were not additive with those of the P301S MAPT transgene (Figures 3C,D). Similar results were observed for astrocytosis (labeled by astrocytic marker GFAP), along with strong co-localization with the Aβ plaques (Figures 3A,D).

The imaging studies of Iba1 convey what occurs locally around neuritic plaques and MAPT pathology, but do not convey the distribution of inflammation in the APP^NL-G-F^/MAPT^P301S^ mouse brain. We performed clarity on APP^NL-G-F^/MAPT^P301S^ mouse brain and WT brain, and then labeled with antibodies to Aβ (4G8), phospho-202/5 MAPT (AT8) and Iba1 (Yang et al., 2014; Ueda et al., 2020). Neuritic plaques were evident throughout the neocortex, as expected (Supplementary Videos S1–3 Aβ = Green). As expected, WT brain exhibited very little labeling (Supplementary Video S3). Labeling for phospho-tau encountered technical difficulties, so was weak, but the labeling that was present in the APP^NL-G-F^/MAPT^P301S^ mouse brains occurred in the hippocampus and entorhinal cortex (Supplementary Videos S2, 3, pS202/5 MAPT, AT8 = Red). These patterns of labeling or Aβ and MAPT are consistent with prior studies of APP^NL-G-F^ and P301S MAPT mice (Yoshiyama et al., 2007; Saito et al., 2014; Sasaguri et al., 2017; Apicco et al., 2018). The MAPT pathology in the APP^NL-G-F^/P301S MAPT mice was much more abundant in the hippocampus and entorhinal cortex than in the neocortex, and the Aβ pathology was greatest in the neocortex. The distribution of microglia was labeled with anti-Iba1 (Supplementary Videos S2, 3, Pink). Interestingly, the Iba1 labeling was much more evident in the hippocampus than in the cortex. The pattern of migration of Iba1 labeling suggests that microglia are more responsive to MAPT pathology than Aβ pathology in the APP^NL-G-F^/P301S MAPT mice.

(Note that technical damage to the brain of APP^NL-G-F^/P301S MAPT mouse #2 (Supplementary Video S3) in the ventral temporal cortex, near the entorhinal region, during removal appeared to elicit strong inflammatory reactivity; we focused less on this reactivity because the relative roles of Aβ/MAPT pathology versus brain damage are difficult to determine.)

To examine the microglia and astrocyte activation through independent approaches, we measured transcripts coding for pro-inflammatory cytokines, anti-inflammatory cytokines and complement proteins in the brain tissue of WT, APP^NL-G-F^, MAPT^P301S^, and APP^NL-G-F^/MAPT^P301S^ mice, respectively (Figures 3E–H). Our result show similar results for multiple arms of the inflammatory cascade. The pro-inflammatory cytokines TNF-α and IL-1β both showed strongest increases in mice carrying the APP^NL-G-F^ gene (Figures 3E,H); interestingly, mice carrying the MAPT^P301S^ and APP^NL-G-F^/MAPT^P301S^ genes exhibited levels that were elevated compared to WT mice, but significantly less than that in the APP^NL-G-F^ mice (Figures 3E,H). A similar pattern was observed for TREM2, a key member of the DAM response, which is thought to participate in epherocytosis (Ulland and Colonna, 2018). Next we examined the complement protein, C1q, which contributes to synaptic pruning and also binds to fibrillar Aβ in AD (Afagh et al., 1996; Rupprecht et al., 2021). In the APP^NL-G-F^/MAPT^P301S^ mouse model, we examined the C1qa RNA level by RT-qPCR and found that it was elevated equally among the APP^NL-G-F^, MAPT^P301S^, and APP^NL-G-F^/MAPT^P301S^ mouse lines compared to WT control (Figure 3F). Finally, we also examined brain-derived neurotrophic factor (BDNFs), which maintains synaptic plasticity and has attracted increasing attention for its potential as a biomarker or therapeutic molecule for AD (Bathina and Das, 2015). Quantification of BDNF transcript by RT-PCR in the APP^NL-G-F^/MAPT^P301S^ mouse model showed elevation BDNF each of the mouse models (Figure 3I).

The combined data indicate that the APP^NL-G-F^/MAPT^P301S^ mouse model recapitulates many of the pivotal neuroinflammatory features of AD, and also suggest that the accumulation of Aβ is a stronger driver of microglial activation and astrogliosis that MAPT pathology.

### 3.4 APP^NL-G-F^ potentiates neurodegeneration in the APP^NL-G-F^/MAPT^P301S^ mice

**Figure 4:**
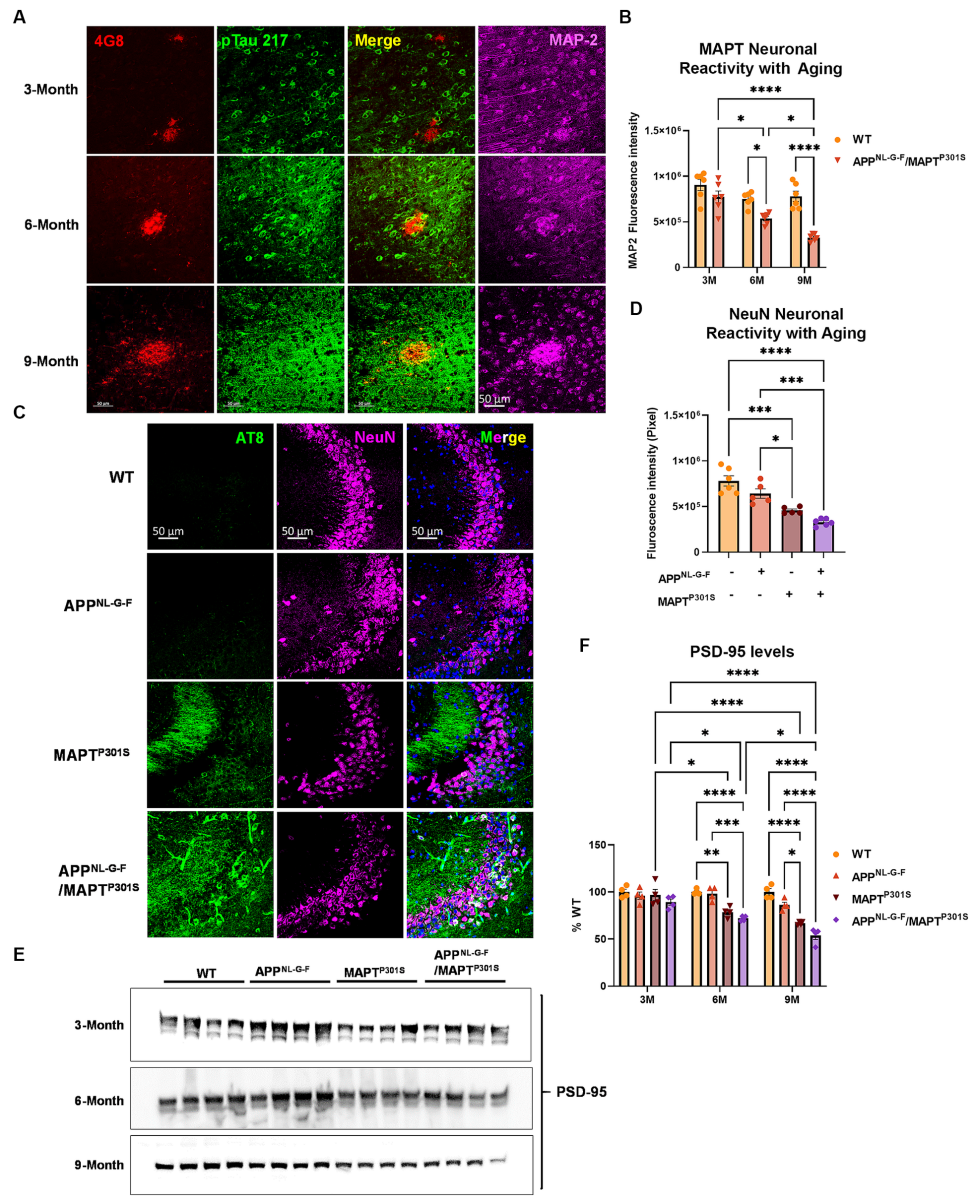
APP^NL-G-F^ potentiates neurodegeneration in the APP^NLGF^/MAPT^P301S^ double transgenic mice. **(A)** Representative images showed the enhanced neurodegeneration (MAP-2, magenta) associated with progressive Aβ deposition (4G8, red) and phosphorylated tau (pTau217, green) accumulation in the APP^NL-G-F^/MAPT^P301S^ mouse brain. Scale bar 50 μm. **(B)** Quantification of neurodegeneration by MAP-2 intensity as shown in **(A)**. *N* = 6 mice in each group; data is shown as mean ± SEM. Two-way ANOVA was used for statistics followed by post hoc analysis with Tukey’s multiple comparisons test, ^*^p < 0.05 and ^****^p < 0.001. **(C)** Representative images showed enhanced neurodegeneration in APP^NL-G-F^/MAPT^P301S^ mouse brain compared to APP^NL-G-F^ or MAPT^P301S^ mouse lines at 9 months. Scale bar 50 μm. **(D)** Quantification of neurodegeneration by NeuN positive neuronal intensity as shown in panel **(C)** (magenta panels). *N* = 6 mice in each group, data is shown as mean ± SEM. One-way ANOVA was used for statistics followed by post hoc analysis with Tukey’s multiple comparisons test, ^*^p < 0.05 and ^***^p < 0.005. **(E,F)** Immunoblot of post-synaptic marker PSD-95 showed the potentiated neurodegeneration in the APP^NL-G-F^/MAPT^P301S^ mouse brain compared to APP^NL-G-F^ or MAPT^P301S^ mouse lines over a 3, 6, and 9 months old. Quantification of band intensities showed that progressive and enhanced decrease of PSD-95 in the APP^NL-G-F^/MAPT^P301S^ mouse brain. *N* = 4 mice in each group, data was normalized to percentage of WT control and is shown as mean ± SEM. Two-way ANOVA was used for statistics followed by post hoc analysis with Tukey’s multiple comparisons test, ^*^p < 0.05, ^**^p < 0.01, ^***^p < 0.005, and ^****^p < 0.001.

Analysis of glial activation in the APP^NL-G-F^/MAPT^P301S^ mouse model prompted us to quantify how expression of neuronal and synaptic markers change with aging in each mouse line (APP^NL-G-F^, MAPT^P301S^, and APP^NL-G-F^/MAPT^P301S^ vs. WT control). Analysis of MAP2 and NeuN showed that the APP^NL-G-F^/MAPT^P301S^ mouse line displayed progressive age-dependent neuronal loss from 3 to 6 and 9 months-old (Figures 4A–D); importantly, the APP^NL-G-F^/MAPT^P301S^ cross showed greater reduction of signal than either the APP^NL-G-F^ and MAPT^P301S^ lines alone, indicating enhanced neurodegeneration for the APP^NL-G-F^/MAPT^P301S^ line. For instance, at 6 months, the APP^NL-G-F^/MAPT^P301S^ mouse showed ~35% loss of MAP-2 compared to WT control, which progressed to more than 60% loss of MAP-2 at 9 months (Figure 4B). In addition to the immuno-fluorescence labeling, we also examined synaptic levels using immunoblot quantification of the post-synaptic marker PSD-95 (Figures 4E,F). The results paralleled those observed for MAP-2 and NeuN, with all KI or transgenic lines exhibiting age-dependent synaptic loss, and the APP^NL-G-F^/MAPT^P301S^ line exhibiting significantly more loss than that observed with either human gene alone (Figures 4E,F).

### 3.5 N^6^-methyl-adenosine and its regulatory enzyme proteins are dysregulated in the APP^NLGF^/MAPT^P301S^ double transgenic mice

N^6^-methyl-adenosine (m^6^A) is the most abundant modification in eukaryotic RNA (Jiang X. et al., 2021). In the recent studies, our group used immunohistochemical labeling of m^6^A to show that m^6^A accumulation is a general feature of AD pathology (Jiang L. et al., 2021). Levels of m^6^A modifications are controlled by addition of m^6^A modifications with m^6^A methyltransferases (also known as writers), such as METTL3/14/16, RBM15/15B and WTAP; m^6^A levels are also controlled by removal via demethylases (also known as erasers), including FTO and ALKBH5 (Flamand et al., 2023). The m^6^A-binding proteins YTHDF1/2/3, YTHDC1/2 IGF2BP1/2/3 and HNRNPA2B1 recognize the modifications; these are also known as “readers” (Jiang X. et al., 2021). Previously we showed that interaction of MAPT with HNRNPA2B1 and m^6^A RNA mediates the progression of tauopathy (Jiang L. et al., 2021). Other studies of AD indicate that m^6^A dysregulation often occurs in the context of altered expression of m^6^A writers and readers (Han et al., 2020; Huang et al., 2020; Deng et al., 2021; Zhao et al., 2021; Zhang et al., 2023). Hence, we examined levels of m^6^A writers and readers in the APP^NL-G-F^/MAPT^P301S^ mouse model.

**Figure 5:**
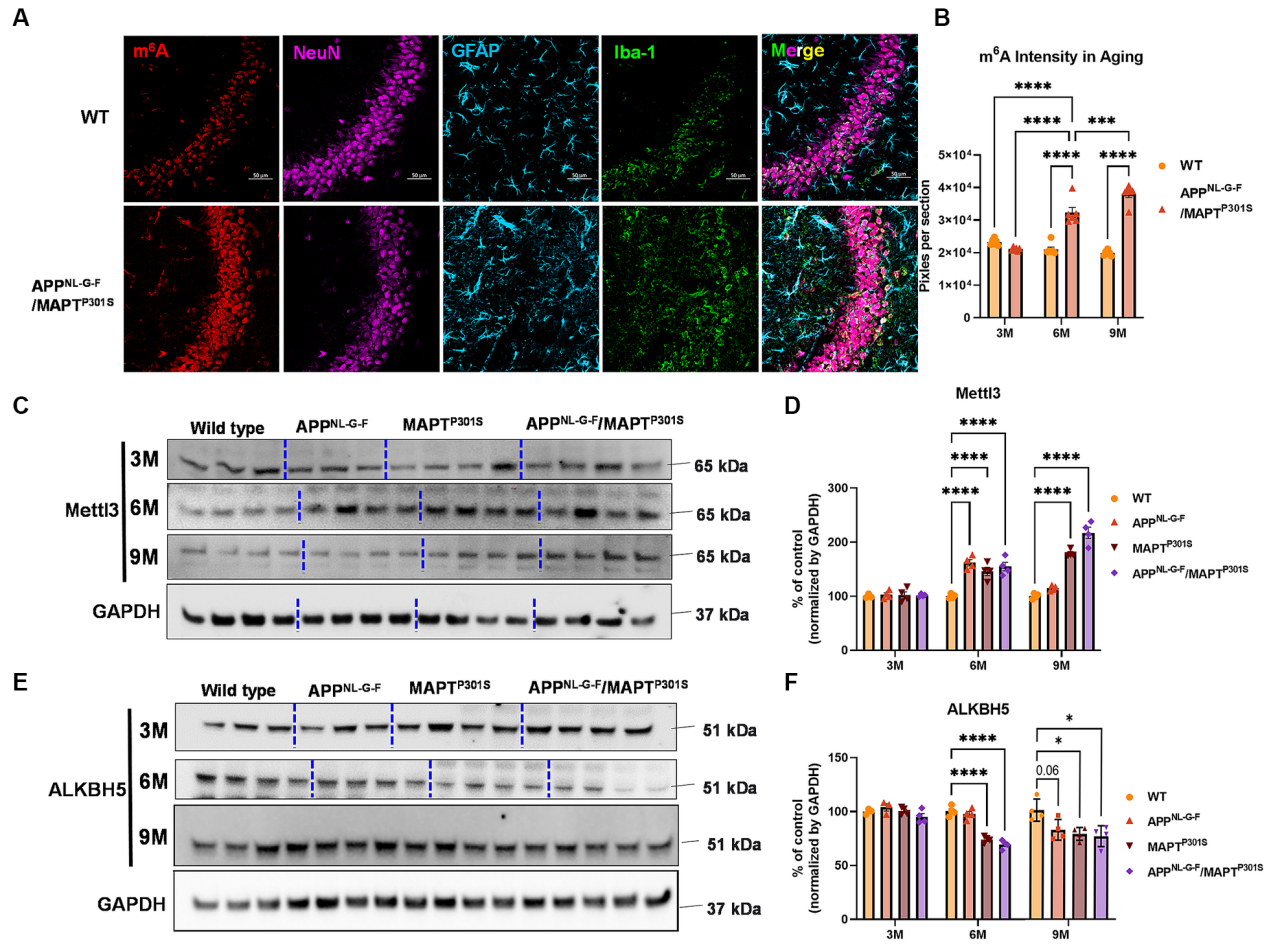
m^6^A and its regulator enzyme proteins are dysregulated in the APP^NLGF^/MAPT^P301S^ double transgenic mice in correspondence to the progression of tau pathology. **(A)** Representative images showed the increased m^6^A intensity in APP^NLGF^/MAPT^P301S^ mouse brain at 6 months compared to WT control. Scale bar 50 μm. **(B)** Quantification of m^6^A intensity in comparison between APP^NLGF^/MAPT^P301S^ and WT control during the aging process. *N* = 6 mice in each group, 3 brain sections were selected from each mouse brain with same position of hippocampus CA3. Data are shown as mean ± SEM. Two-way ANOVA was used for statistics followed by post hoc analysis with Tukey’s multiple comparisons test, ^***^p < 0.005 and ^****^p < 0.001. **(C,D)** Immunoblot analysis of the m^6^A methyltransferase Mettl3 showed progressively increased intensity in APP^NL-G-F^ /MAPT^P301S^ mouse brain compared to APP^NL-G-F^ or MAPT^P301S^ alone at 3, 6, and 9 months. GAPDH was detected and used as internal control for statistical analysis. *N* = 4 mice in each group, data was normalized to percentage of WT control and is shown as mean ± SEM. Two-way ANOVA was used followed by post hoc analysis with Tukey’s multiple comparisons test, ^****^p < 0.001. **(E,F)** Immunoblot analysis of the m^6^A RNA demethylase ALKBH5 showed decreased intensity in APP^NL-G-F^/MAPT^P301S^ mouse brain compared to APP^NL-G-F^ or MAPT^P301S^ alone during the aging process at 3 to 6 and 9 months. Quantification of band intensity showed the decreased ALKBH5 correlated with MAPT pathology in APP^NL-G-F^/MAPT^P301S^ and MAPT^P301S^ mouse brain. GAPDH was used as internal control for statistical analysis. *N* = 4 mice in each group, data was normalized to percentage of WT control and is shown as mean ± SEM. Two-way ANOVA was used followed by post hoc analysis with Tukey’s multiple comparisons test, ^****^p < 0.001.

Immunofluorescence studies with anti-m^6^A antibody suggest that total levels of m^6^A progressively increased in the APP^NL-G-F^/MAPT^P301S^ mouse at 3, 6, and 9 months compared to WT control (Figures 5A,B). Immunoblotting show that the m^6^A methyltransferase Mettl3 also increased in the APP^NL-G-F^, MAPT^P301S^ and APP^NLGF^/MAPT^P301S^ compared to WT control at 6 months (Figures 5C,D). However, at 9 months only the MAPT^P301S^ or APP^NLGF^/MAPT^P301S^ mouse lines showed significant increases in Mettl3 levels (Figures 5C,D). The m^6^A eraser ALKBH5 showed a small but significant decrease in expression in MAPT^P301S^ and APP^NLGF^/MAPT^P301S^ mouse compared to WT control at 6 and 9 months (Figures 5E,F). These results demonstrate dysregulation of m^6^A and enzymes in the m^6^A pathway in a manner similar to that observed in AD, and suggest that the phenomenon is predominantly driven by MAPT^P301S^ tau pathology.

## 4 Discussion

The results presented above show that Aβ enhances tau pathologies (phosphorylation, misfolding, and fibrillization) in the context of P301S MAPT over-expression; our interpretation of the results is summarized in Graphical abstract. Neurodegeneration parallels tau pathology, being enhanced in the double transgenic mouse. However, the converse is not true. Aβ pathologies (neuritic plaque, Aβ_40_ and Aβ_42_ load) are not greater in the APP^NL-G-F^/MAPT^P301S^ mouse than in the APP^NL-G-F^ mouse alone. These results are consistent with those recently reported for an APP^NL-G-F^/MAPT^P290S^ mouse, which is a mouse model in which both human APP and human MAPT are knockins (Huang et al., 2022). The report on the APP^NL-G-F^/MAPT^P290S^ mouse showed enhancement of MAPT pathology by Aβ but the pathology occurs only in very old mice, and the manuscript did not provide any information on other pathologies, such as inflammation or m^6^A. We now report that inflammation occurs near Aβ pathology, but the gross distribution of inflammation reflects the gross distribution of tau pathology. In addition, the amount and distribution of m^6^A parallels MAPT pathology.

Our results for Aβ and MAPT pathologies are generally consistent with prior observations using crosses of APP over-expression mouse lines and MAPT over-expression mouse models (Saul et al., 2013; Chabrier et al., 2014; Heraud et al., 2014; Stancu et al., 2014; Chen et al., 2016; Kang et al., 2021). These over-expression models consistently observe that the presence of Aβ pathology enhances the accumulation of MAPT pathology, neurodegeneration and cognitive loss (Saul et al., 2013; Chabrier et al., 2014; Heraud et al., 2014; Stancu et al., 2014; Chen et al., 2016; Kang et al., 2021). These same studies indicate that the presence of MAPT pathology either does not change the accumulation of Aβ pathology or decreases it (Saul et al., 2013; Chabrier et al., 2014; Heraud et al., 2014; Stancu et al., 2014; Chen et al., 2016; Kang et al., 2021), with only one initial study suggesting that MAPT pathology increases Aβ pathology (Ribe et al., 2005). The enhancement of MAPT pathology in the APP^NL-G-F^/MAPT^P301S^ mouse is also evident in the distribution of MAPT pathology in the hippocampus. The APP^NL-G-F^ model exhibits modest phospho-MAPT pathology at 6 and 9 months that is evident in the dendritic fields in the CA3 region, while the APP^NL-G-F^/MAPT^P301S^ cross exhibits MAPT pathology in the dendritic fields as well as in the neuronal soma of CA3 (Figure 4D). The cell body MAPT pathology is particularly important because this pathology colocalizes with markers of the translational stress response, such as stress granule proteins HNRNPA2B1, TIA1, EIF3n and PABP, and also neuronal death (as shown by cleaved caspase 3 and loss of NeuN positive cells) (Ash et al., 2014; Apicco et al., 2018; Maziuk et al., 2018; Apicco et al., 2019; Jiang et al., 2019, 2021). Thus, neurodegeneration appears to be potentiated in the APP^NL-G-F^/MAPT^P301S^ model.

The distributions of the Aβ and MAPT pathologies observed in the APP^NL-G-F^/MAPT^P301S^ model are notably distinct. The classic model of AD is based on the Aβ cascade and proposes that aggregated Aβ stimulates inflammation, and that these two factors produce additive injury to elicit neurodegeneration in AD (Scheltens et al., 2021). This injury is further enhanced by aging, vascular damage, metabolic conditions such as Type II Diabetes and genetic risk factors (Scheltens et al., 2021). The direct connection between Aβ and MAPT pathology is poorly understood, as shown by the weak correlation between the distribution of Aβ pathology and the distribution of MAPT pathology in humans (Serrano-Pozo et al., 2011; Perez-Nievas et al., 2013; Scheltens et al., 2021). The distribution of pathology in the APP^NL-G-F^/MAPT^P301S^ model highlights the poor spatial correlation between these pathologies. The differential distribution of the pathologies might be further accentuated by the strong genetic factors driving the accumulation of MAPT pathology in this model (Graphical abstract). MAPT pathology in the MAPT^P301S^ model accumulates so rapidly because of the strong MAPT expression (the human protein is expressed about 5-fold higher than the endogenous mouse protein) and the protein itself has a mutation that enhances the rate of aggregation (Yoshiyama et al., 2007). These two factors present a strong force driving MAPT aggregation (Graphical abstract). Such a strong genetic push reduces the need for a toxin, such as Aβ, to drive the MAPT pathology, although the accumulation of Aβ in the APP^NL-G-F^/MAPT^P301S^ model clearly does accelerate the appearance and enhance the amount of MAPT pathology moderately (Graphical abstract). From an anatomical perspective, the disparate distribution of Aβ and MAPT pathology in the APP^NL-G-F^/MAPT^P301S^ model might also be accentuated by the strong genetic engineering that drives the MAPT pathology, although general toxicity from Aβ accumulation does appear to enhance total MAPT pathology and neurodegeneration in this model.

Increasing evidence points to a key role for inflammation in AD. Many AD-linked genes appear to enhance disease risk by affecting the biology of microglia. For instance, TREM2 is one of the strongest risk factors for AD, and evidence suggests that TREM2 acts to recognize Aβ pathology and direct microglial responses toward the pathology (Guerreiro et al., 2013; Zhou et al., 2020). The strong impact of Aβ on inflammation is also evident in the APP^NL-G-F^/MAPT^P301S^ model. Inflammatory cells are readily evident in the area around Aβ plaques (Figure 3A). These microglia were more abundant and showed greater ramifications than in the P301S MAPT model. However, it is important to note that microglia do respond to MAPT pathology, Iba-1 reactivity was increased in the P301S MAPT model, but not to the same level as observed in the APP^NL-G-F^ or APP^NL-G-F^/MAPT^P301S^ model. The inflammatory responses (Figure 3) are interesting when compared to the neurodegenerative responses (Figure 4). As mentioned, inflammatory responses are higher for the APP^NL-G-F^ than the MAPT^P301S^ or the APP^NL-G-F^/MAPT^P301S^ models. However, neurodegeneration is greater for the APP^NL-G-F^/MAPT^P301S^ model that the APP^NL-G-F^ or MAPT^P301S^ models. The differential sensitivities of inflammation and neurodegeneration suggest that MAPT is a stronger driver of neurodegeneration than is inflammation in this animal model (Graphical abstract). Thus, while the “Disease Associated Microglial” phenotype is strongly associated with disease and might be an important driver of disease in humans, our results suggest that in the APP^NL-G-F^/MAPT^P301S^ mouse model MAPT pathology is a stronger driver of neurodegeneration than inflammation. These results are also consistent with published work indicating that Aβ pathology strongly stimulates the “Disease Associated Microglial” phenotype while MAPT pathology elicits microglial responses exhibiting weaker cytokine production (Paolicelli et al., 2022).

The increases observed for m^6^A parallel those reported by our group previously (Jiang L. et al., 2021). Other groups have also observed that m^6^A accumulates in models of tauopathy, as well as in other diseases with intracellular aggregates, including ALS and Huntington’s disease (McMillan et al., 2023; Nguyen et al., 2023; Atrian et al., 2024). Interestingly, m^6^A does not appear to accumulate in models of Aβ amyloidosis, which accumulate aggregates extracellularly (Han et al., 2020). These models develop little intracellular MAPT pathology, nor other types of neuronal protein aggregates (Oakley et al., 2006). Mouse MAPT has only 3 repeats and a much lower tendency to aggregate; since aggregated MAPT is thought to mediate Aβ induced degeneration, the absence of MAPT pathology also might explain the low levels of neurodegeneration observed in APP mouse models (Oakley et al., 2006). In the current study, increases in m^6^A were observed most prominently in neurons, and that levels of m^6^A correlated with levels of MAPT pathology. This finding is consistent with the observation that MAPT pathology co-localizes with the RNA binding protein, HNRNPA2B1, which functions as an indirect m^6^A reader. The increases in m^6^A are also consistent with the observed increase in the m^6^A writer, METTL3, and decrease in m^6^A eraser, ALKBH5 (Tong et al., 2018).

Our study is designed to probe the link between m^6^A, MAPT and Aβ. Thus, the results presented above in Figure 5 directly compare m^6^A levels in the context of MAPT pathology, Aβ pathology and combined MAPT and Aβ pathology. The results are clear in that m^6^A levels (as determined by immunohistochemistry with the anti- m^6^A antibody) follow MAPT pathology (Graphical abstract). Our prior work shows that oligomeric MAPT (which is highly phosphorylated) forms a complex with m^6^A and the m^6^A reader HNRNPA2B1 (Jiang L. et al., 2021). Multiple groups also have shown that MAPT functions in part to facilitate the integrated stress response and formation of stress granules, as well as to regulate ribosomal function. m^6^A is known to accumulate in the cytoplasm in response to stress, but the function of cytoplasmic m^6^A remains to be clearly delineated (Meier et al., 2016; Briggs et al., 2017; Koren et al., 2019; Ries et al., 2019; Wolozin and Ivanov, 2019; Fu and Zhuang, 2020). The studies in this manuscript indicate that m^6^A responds to MAPT rather than Aβ pathology, although the functions of cytoplasmic m^6^A remains to be determined.

Use of an anti-m^6^A antibody has limitations. The first consideration is specificity. Antibodies against m^6^A also recognize demethylated adenosine, m6Am. This modification constitutes 2–10% of total m^6^A detected in tissues, with brain being on the lower end of the abundance (Linder et al., 2015). Interestingly, m^6^Am also non-enzymatically converts to m^6^A, so the biological impact of m^6^Am is currently ambiguous (Linder et al., 2015; Flamand et al., 2023). A second consideration is the nature of the RNA species showing the increase. The m^6^A modification occurs in all types of RNA, although it has the lowest abundant in tRNA (Linder et al., 2015; Flamand et al., 2023). Use of the m^6^A antibody does not provide insight into methylation of individual RNA molecules. m^6^A RNAseq approaches are required to provide mechanistic understanding of the changes in methylation and to identify specific molecules (e.g., mRNA transcripts) whose methylation impacts on disease.

Our studies also provide insight into potential mechanisms regulating m^6^A in disease. The increases in m^6^A observed correlate with MAPT pathology are show a corresponding association with increased levels of METTL3 and reduced levels of ALKBH5, which are the enzymes that, respectively, add and remove m^6^A from mRNA. More writing combined with reduced erasure leads to increased levels of m^6^A. Although changes in m^6^A were most evident in neurons, the results in Figure 5A show that some astrocytes and microglia also exhibited strong increases in m^6^A in the APP^NL-G-F^/MAPT^P301S^ model. Such results are consistent with emerging studies showing that m^6^A regulates inflammation, macrophages and also astrocytosis (Cockova et al., 2021; Yi et al., 2021; Sun Z. et al., 2022; Sun J. et al., 2022; Wang et al., 2022). Such findings raise the possibility that m^6^A modulation might also be applied towards regulation of inflammation in AD; indeed a recent study observed that conditional knockout of METTL3 in microglia attenuated inflammation and Aβ accumulation in a mouse model based on Aβ injection (Yin et al., 2023).

Another final important consideration is the type of AD model. Total m^6^A levels were also reported in a previous manuscript examining m^6^A in an AD model utilizing over-expression of only APP, which is a model that exhibits Aβ accumulation without corresponding MAPT pathology (Han et al., 2020). Data from this manuscript suggest that the absence of MAPT pathology in this model produced a corresponding absence of m^6^A accumulation (Han et al., 2020).

## 5 Conclusion

The field is increasingly moving towards use of KI models. The APP^NL-G-F^/MAPT^P301S^ model described in this manuscript takes advantage of the APP^NL-G-F^ KI line, but utilizes the human MAPT^P301S^ line in order to achieve robust MAPT pathology. Our results show that this model provides an appealing alternative to the double APP^NL-G-F^ × MAPT KI model, which develop both MAPT pathologies very slowly (Saito et al., 2019). The current APP^NL-G-F^/MAPT^P301S^ model thus provides a useful compromise. This model exhibits strong Aβ accumulation and strong inflammation, while avoiding artifacts associated with APP or presenilin over-expression. The APP^NL-G-F^/MAPT^P301S^ model also possesses the benefits arising from strong expression of human MAPT with the resulting rapid development of robust MAPT pathology, strong m^6^A accumulation and, importantly, significant neurodegeneration.

## Data availability statement

The raw data supporting the conclusions of this article will be made available by the authors, without undue reservation.

## Ethics statement

The animal studies were approved by Boston University Institutional Animal Care and Use Committee. The studies were conducted in accordance with the local legislation and institutional requirements.

## Author contributions

LJ: Conceptualization, Formal analysis, Methodology, Writing – original draft, Writing – review & editing, Data curation, Investigation, Validation. RR: Investigation, Methodology, Writing – review & editing. MW: Investigation, Methodology, Writing – review & editing. LZ: Investigation, Methodology, Writing – review & editing. CW: Formal analysis, Investigation, Methodology, Writing – review & editing. JL: Formal analysis, Writing – review & editing. ZW: Formal analysis, Writing – review & editing. AK: Investigation, Writing – review & editing. MJ: Investigation, Writing – review & editing. AO: Formal analysis, Investigation, Methodology, Writing – review & editing. LD: Investigation, Methodology, Writing – review & editing. JS: Investigation, Writing – review & editing. GS: Investigation, Writing – review & editing. SR: Investigation, Writing – review & editing. CK: Formal analysis, Writing – review & editing. SD: Investigation, Writing – review & editing. PD: Conceptualization, Formal analysis, Writing – review & editing. BN: Formal analysis, Writing – review & editing. WX: Writing – review & editing. TSait: Resources, Writing – review & editing. TSaid: Resources, Writing – review & editing. BW: Conceptualization, Formal analysis, Funding acquisition, Methodology, Project administration, Resources, Supervision, Writing – original draft, Writing – review & editing.
